# Impact on mortality following first acute myocardial infarction of distance between home and hospital: cohort study

**DOI:** 10.1136/hrt.2007.123612

**Published:** 2007-11-05

**Authors:** L Wei, C C Lang, F M Sullivan, P Boyle, J Wang, S D Pringle, T M MacDonald

**Affiliations:** 1 Medicines Monitoring Unit (MEMO), Division of Medicine and Therapeutics, Ninewells Hospital and Medical School, Dundee, UK; 2 Division of Medicine and Therapeutics, Ninewells Hospital and Medical School, Dundee, UK; 3 Tayside Centre for General Practice, Division of Community Health Sciences, Dundee, UK; 4 School of Geography and Geosciences, University of St Andrews, St Andrews, UK

## Abstract

**Objective:**

To investigate the effect of distance between home and acute hospital on mortality outcome of patients experiencing an incident myocardial infarction (MI).

**Design:**

Cohort study using a record linkage database.

**Setting:**

Tayside, Scotland, UK.

**Patients:**

10 541 patients with incident acute MI between 1994 and 2003 were identified from Tayside hospital discharge data and from death certification data.

**Main outcome measures:**

MI mortality in the community, all-cause mortality in hospital and all-cause mortality during follow-up.

**Results:**

4133 subjects died following incident MI in the community (that is, were not hospitalised), 6408 patients survived to be hospitalised and 1010 of these (15.8%) died in hospital. Of 5398 discharged from hospital, 1907 (35.3%) died during a median of 3.2 years of follow-up. After adjustment for rurality and other known risk factors, distance between home and admitting hospital was significantly associated with increased mortality both before hospital admission (adjusted odds ratio (OR), 2.05, 95% CI 1.00 to 4.21 for >9 miles and 1.46, 1.09 to 1.95 for 3–9 miles when compared to <3 miles) and after hospitalisation (adjusted hazard ratio (HR) 1.90, 1.19 to 3.02 and 1.27, 0.96 to 1.68). However, there was no effect of distance on in-hospital mortality (adjusted OR 0.95, 0.45 to 2.03 and 1.02, 0.66 to 1.58).

**Conclusion:**

The distance between home and hospital of admission may predict mortality in subjects experiencing a first acute MI. This association was found both before and after hospitalisation. Further studies are needed to explore the reasons for this association. However these data provide support for policies that locate services for acute MI closer to where patients live.

Geographical inequalities in care in the UK National Health Service (NHS) may relate to the physical distance between patients and NHS facilities. In Scotland, official policy in the government’s response to the Kerr report, supports the greater provision of acute services in the community.^1^ ^2^ Geographical inequality for patients with myocardial infarction (MI) may be related to time to thrombolysis as it is established that early thrombolysis and cardiopulmonary resuscitation improve the mortality outcome.^3^ ^4^ Geographical inequality could also be explained by more stoical behaviour of subjects in a rural setting or other factors. We have done a large population-based study to investigate the effect of distance between home and acute hospital on mortality outcome of patients experiencing an incident MI.

## METHODS

The study was carried out in Tayside, Scotland, using the Medicines Monitoring Unit’s record-linkage database. The database covers a population of approximately 400 000 within a geographical area of approximately 4600 square miles. The data collection methods for this database have previously been described.^5^ In brief, it contains several datasets including all dispensed community prescriptions, hospital discharge data, biochemistry results and other datasets that are linked by a unique patient identifier, the community health number. Diagnoses have been validated by inspection of the general practitioner (GP) records.^6^ These data are anonymised for the purposes of research. The project was approved by the Tayside Caldicott Guardians who are appointed by the government to protect the confidentiality of medical records and the Tayside committee on research medical ethics.

### Patient population

The study population included subjects who were resident in Tayside and registered with a GP in January 1994 and remained in Tayside until December 2003 or died during the study period—a fixed population (n = 347 131). Study subjects were patients experiencing an incident MI who had not previously been hospitalised with a diagnosis of acute MI between January 1994 and December 2003.

#### Prehospital coronary mortality

We studied patients who died out-of-hospital with a primary certified cause of death (data from the General Register Office for Scotland) of MI (ICD-9 code 410 and ICD-10 code I21).

#### Patients hospitalised with MI and follow-up mortality

Patients admitted to Tayside hospitals with their first MI were identified from the Tayside hospital discharge data using the primary diagnosis ICD-9 code of 410 and ICD-10 code I21. We tracked the all-cause mortality of patients during their hospital stay and following discharge from hospital.

### Distance between home and hospital of admission

Distance between home and hospital of admission was calculated based on the grid reference of the postcode of the patient’s address and the grid reference of the address of the admitting hospital. For patients who died outside hospital the grid reference of their nearest acute hospital was used to calculate the distance between home and the closest possible hospital of admission. The thrombolytic treatment strategy for Tayside during the study period was in-hospital initiation of treatment. About half of the patients lived within 5 miles (8 km) from these hospitals and 98.5% patients lived within 25 miles. The distance between home and hospital was thus categorised into tertiles: (1) distance <3 miles; (2) distance between 3–9 miles; (3) distance >9 miles. We obtained a rurality code from patient postcodes (that is, urban and rural classification of postcodes for Scotland, a code of 1 means large urban area with settlement of over 125 000 people and a code of 8 means very remote rural area with settlement of fewer than 3000 people with a drive time of over 60 minutes to a settlement of 10 000 or more).^7^ We also calculated the travel time by car along the road network, the car speed being based on the speed limit for the different types of road.

### Thrombolysis data

Three major hospitals (Ninewells Hospital, Perth Royal Infirmary and Stracathro Hospital) in Tayside provided acute services. All these hospitals had coronary care units and accredited cardiologists working in them. However, catheter laboratory facilities were available only at Ninewells Hospital. In-hospital thrombolysis data were available for Ninewells Hospital between December 2001 and December 2004 (n = 366). We used these data in a subgroup analysis of patients to access the impact of thrombolysis on outcome.

### Postmortem examination for community MI death

Data were obtained from the General Register Office for those patients who had been certified as an MI death in the community and who also had a subsequent postmortem examination. These subjects were more likely to be those in whom there was some diagnostic uncertainty and many of these post mortems were carried out for medicolegal reasons.

### Study outcome

The outcome was the individual components: prehospital MI mortality, in-hospital all-cause mortality and follow-up all-cause mortality until 31 December 2003.

### Statistical analysis

Patients’ characteristics were summarised as mean (SD) for continuous variables and number of subjects (%) for categorical variables. The χ^2^ and t tests were performed to determine significant differences between dead and alive patients. The Scottish 2000 standard population was used to calculate the age-standardised mortality rates. A logistic regression model was used to estimate the effects of distance between home and hospital of admission for both prehospital mortality and in-hospital mortality. Survival analysis was used to estimate the effects of distance between home and hospital of admission for post-hospital mortality since patients had different follow-up times. The results were adjusted for demographic information including age, gender, Carstairs deprivation score (derived from the patients’ postcode and census data comprising social class, overcrowding, male unemployment and non-car ownership^8^), other covariates including day of week of admission,^9^ diabetes mellitus, and cardiovascular drug use (lipid-lowering drugs, antiplatelet drugs, β-blockers, angiotensin-converting enzyme (ACE) inhibitors, diuretics, nitrates, α-blockers, calcium blockers), interactions between distance and social deprivation or gender and estimated travel time and rurality. All statistical analyses were carried out using SAS version 8.2 (SAS institute, Cary, NC, USA).

## RESULTS

**Figure 1:**
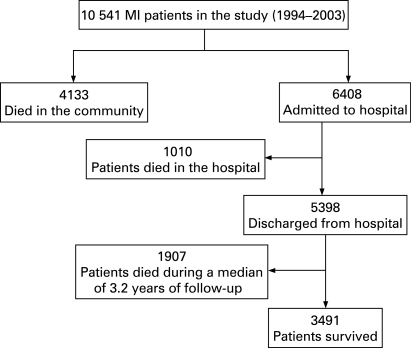
Flow chart of patients in the study.

This study included 10 541 patients (fig 1). Table 1 shows the characteristics of the patients. The estimated median travel times by car from home and admitted hospital were 5.9 minutes (interquartile range (IQR) 3.3–8.9), 15.2 minutes (IQR 11.4–18.2) and 37.1 minutes (IQR 30.2–41.1) for the short, medium and long-distance groups, respectively.

**Table 1:** Characteristics of patients following myocardial infarction, Tayside

	Prehospital		In-hospital				Post-hospital			
	Dead		Dead		Alive		Dead		Alive	
	No	%	No	%	No	%	No	%	No	%
Distance (miles)†										
<3	1287	31.7	407	41.6	1753	33.0**	678	36.5	1075	31.1**
3–9	1346	33.2	304	31.0	1801	33.9	607	32.7	1194	34.6
>8	1423	35.1	268	27.4	1756	33.1	572	30.8	1184	34.3
Sex										
Male	2029	49.1	443	43.9	3270	60.6**	1017	53.3	2253	64.5**
Female	2104	50.9	567	56.1	2128	39.4	890	46.7	1238	35.6
Age (mean, SD)	77.2	10.9	78.5	9.6	67.5	12.6**	74.6	10.2	63.7	12.1**
Deprivation category†										
1, least deprived	175	4.4	47	4.8	313	5.9	100	5.4	213	6.1
2	833	20.9	172	17.4	980	18.4	325	17.6	655	18.8
3	1187	29.8	312	31.6	1508	28.3	502	27.3	1006	28.8
4	424	10.7	122	12.3	561	10.5	216	11.7	345	9.9
5	487	12.2	111	11.2	648	12.2	221	12.0	427	12.2
6+, most deprived	876	23.0	225	22.8	1320	24.8	478	26.0	742	24.1
Travel time (mean, SD)	–	–	17.3	15.3	19.3	14.9**	20.4	15.7	18.7	15.4**
Urban-rural code‡										
1	1603	40.5	385	38.6	2356	44.0**	813	43.5	1543	44.2
2	1003	25.4	289	29.0	1410	26.3	527	28.2	883	25.3
3, 6	1181	29.9	280	18.0	1431	26.7	467	25.0	964	27.6
7, 8	167	4.2	44	4.4	163	3.0	62	3.3	101	2.9
Previous hospitalisation										
Angina	445	10.8	121	12.0	691	12.8	295	15.5	396	11.3**
Heart failure	845	20.5	277	27.4	1402	26.0**	821	43.1	581	16.6**
Stroke	433	10.5	111	11.0	271	5.0**	173	9.1	98	2.8**
Hypertension	432	10.5	131	13.0	645	12.0*	233	12.2	412	11.8**
PVD	462	11.2	128	12.7	423	7.8**	232	12.2	191	5.5**
Diabetes	567	13.7	146	14.5	715	13.2	327	17.2	388	11.1**
Cardiovascular drug use in the previous year‡										
Lipid-lowering drug	325	7.9	97	9.6	667	12.4*	554	29.1	2660	76.2**
Antiplatelets	1569	38.0	386	38.2	1556	28.8**	1205	63.2	2907	83.3**
β-blockers	1148	27.8	302	29.9	1684	31.2	539	28.3	2264	64.9**
ACE inhibitors	868	21.0	188	18.6	787	14.6**	765	40.1	2054	58.8**
Diuretics	2570	62.2	628	62.2	2110	39.1**	1052	55.2	1388	39.8**
α-blockers	198	4.8	56	5.5	257	4.8**	54	2.8	123	3.5
Nitrates	1438	34.8	374	37.0	1627	30.1**	1047	54.9	2435	69.8**
Ca-blockers	1370	33.2	370	36.6	1694	31.4	518	27.2	1177	33.7**

Data are numbers and percentages unless otherwise stated.

*Comparison of dead and alive patients, p<0.05; **p<0.01. †Excluding missing data. ‡Drug uses during the follow-up for post-hospital cohort.

ACE, angiotensin-converting enzyme; PVD, peripheral vascular disease.

### Prehospital mortality

In all, 4133 patients with MI as the primary certified cause of death died without being hospitalised or before they reached a hospital (table 1). The age-adjusted death rate was 8.4% (8.0% for men and 9.3% for women); 50.9% of patients were women and 35.1% of patients lived >9 miles from the hospital. Compared with patients living <3 miles from hospitals those living >3 miles from hospital had higher MI mortality (adjusted OR 1.46, 95% CI 1.09 to 1.95 for 3–9 miles and 2.05, 1.00 to 4.21 for >9 miles).

#### Diagnostic accuracy of community MI death

About 10% of patients certified as a community MI death had postmortem examinations. The postmortem results showed that MI was the cause of death in over 95% of these subjects during the study period.

### In-hospital mortality

A total of 6408 patients (57.9% male) survived to be hospitalised with incident acute MI during the study period and 1010 of these (15.8%) died in hospital (that is, case fatality). The median duration of hospitalisation was 2 days. Except for β-blocker use patients who died in hospital had received more cardiovascular drug prescriptions in the previous year than patients who survived. There were significant differences in gender, age and social deprivation between patients who survived and those who died. Women and older patients had higher risk of mortality than men and young patients. The age-adjusted death rate was 11.6% (12.3% for men and 10.8% for women). There were more women living closer to hospital than men. The percentages of women in each distance category were 36.2%, 33.1% and 30.6% for <3 miles, 3–9 miles and >9 miles distance groups, respectively.

There was no increased risk of death in hospital with increasing distance between home and hospital of admission (table 2).

**Table 2:** Adjusted odds ratios (OR) or hazard ratios (HR) for mortality in patients with MI

	Prehospital	In-hospital	Post-hospital	
	Number of event/total			
	OR or HR			
	OR (95% CI)	OR (95% CI)	HR (95% CI)	
Distance (miles), tertile*				
<3	1287/3447	407/2160	678/1753	
3–9	1346/3451	304/2105	607/1801	
>9	1423/3447	268/2024	572/1756	
Unadjusted				
<3	1.00	1.00	1.00	
3–9	1.07 (0.97 to 1.18)	0.73 (0.62 to 0.86)	0.83 (0.74 to 0.92)	
>9	1.18 (1.07 to 1.30)	0.66 (0.56 to 0.78)	0.88 (0.79 to 0.98)	
Adjusted for age, sex and deprivation				
<3	1.00	1.00	1.00	
3–9	1.17 (1.05 to 1.30)	0.80 (0.66 to 0.95)	0.89 (0.79 to 1.00)	
>9	1.20 (1.07 to 1.35)	0.63 (0.52 to 0.77)	0.96 (0.86 to 1.10)	
Adjusted for age, sex, deprivation and other covariates				
<3	1.00	1.00	1.00	
3–9	1.16 (1.04 to 1.29)	0.82 (0.68 to 0.98)	0.90 (0.80 to 1.01)	
>9	1.18 (1.05 to 1.32)	0.66 (0.54 to 0.80)	0.97 (0.85 to 1.10)	
Adjusted for age, sex, deprivation, other covariates and urban-rural code				
<3	1.00	1.00	1.00	
3–9	1.21 (1.07 to 1.37)	1.07 (0.73 to 1.58)	1.21 (0.95 to 1.54)	
>9	1.30 (1.10 to 1.54)	0.79 (0.44 to 1.40)	1.49 (1.04 to 2.15)	
Adjusted for age, sex, deprivation, other covariates and estimated travel time				
<3	1.00	1.00	1.00	
3–9	1.44 (1.16 to 1.78)	0.65 (0.44 to 0.98)	1.19 (0.90 to 1.58)	
>9	2.37 (1.24 to 4.54)	1.04 (0.22 to 4.93)	2.06 (1.01 to 4.21)	
Adjusted for age, sex, deprivation, other covariates, urban-rural code and estimated travel time				
<3	1.00	1.00	1.00	
3–9	1.46 (1.09 to 1.95)	1.02 (0.66 to 1.58)	1.27 (0.96 to 1.68)	
>9	2.05 (1.00 to 4.21)	0.95 (0.45 to 2.03)	1.90 (1.19 to 3.02)	

*Other covariates included day of week of admission, previous cardiovascular disease, diabetes mellitus, cardiovascular drug use and interactions between distance and deprivation/sex. Analysis was done by excluding missing data.

### Follow-up mortality

In all, 5398 patients (3260 men, 2128 women) were discharged from hospital and were followed up for a median of 3.2 years (IQR 1.1–6.2). Of these, 1907 patients (35.3%, 1017 men and 890 women) died during the follow-up period. The age-adjusted death rate was 8.6% (8.4% for men and 9.4% for women). There was a significantly increased use of statins and other cardiovascular drugs following hospitalisation compared with previous drug use (table 1). Patients who survived during follow-up had higher prescribing rates for cardiovascular drugs than patients who died (as we have shown before).^10^ A total of 3214 patients (59.5%) were taking lipid-lowering drug treatment and, of these, 3194 (99.4%) were taking statin treatment.

After adjustment for other risk factors, patients who lived >9 miles from the hospital had an increased risk of mortality during the follow-up period when compared to patients who lived <3 miles from the hospital (adjusted HR 1.90, 95% CI 1.19 to 3.02) (table 2). A non-significant increased mortality was seen for those who lived 3-9 miles from the hospital (adjusted HR 1.27, 95% CI 0.96 to 1.68).

#### Effect of cardiovascular drugs

Compared with non-users, users of lipid-lowering drugs (adjusted HR 0.47, 95% CI 0.42 to 0.53), antiplatelet drugs (adjusted HR 0.40, 95% CI 0.35 to 0.45), β-blockers (adjusted HR 0.55, 95% CI 0.49 to 0.62) and ACE inhibitors (adjusted HR 0.84, 95% CI 0.75 to 0.93) during the follow-up had a significantly lower risk of mortality.

#### Thrombolysis

A total of 209 out of 5398 (3.8%) patients had thrombylosis data for the follow-up analysis. This subgroup analysis suggested that early thrombolysis reduced mortality (adjusted HR 0.63, 95% CI 0.37 to 1.07), However, early thrombolysis did not change the direction of the impact of distance (adjusted HR 1.86, 95% CI 0.49 to 7.02 for patients who lived 3–9 miles from the hospital, adjusted HR 1.43, 95% CI 0.18 to 11.39 for patients who lived >9 miles from the hospital when compared with patients who lived <3 miles from the hospital).

## DISCUSSION

Previous studies have shown a relation between the treatment delay and mortality outcome in MI patients.^11^^–^14 However, few studies have investigated the impact of geographical location and none has been representative of a complete population.^15^ Our data suggest that the distance between home and hospital of admission predicts mortality in subjects experiencing first acute MI both in the community (prehospital) and following discharge from hospital after adjustment for urban-rural code or travel time and other covariates. There was no difference if these results were adjusted only for age, sex and other covariates, suggesting that urban-rural code and travel time are the significant confounders. Other areas that could cause a delay between the onset of symptoms and treatment are the delay by patients in calling for help and the delay between calling for help and the attendance of a doctor. We could not measure these but they could be investigated.^16^ However, we used a measure of rurality as a possible surrogate measure of patient behaviour in the different geographical settings.

The median travel time was 37 minutes for patients who lived >9 miles from an acute hospital. This time does not include time taken for the ambulance to reach the patient. In addition, these times take no account of adverse traffic or weather conditions. We think that it is unlikely that this group of patients could have met the national target for thrombolysis within 60 minutes of a call for help.

### Geographical inequality and early thrombolysis

Our data support the concept that there is geographical inequality of care in patients who have had an acute incident MI. An excess in coronary heart disease (CHD) death rates in young patients was reported outside the state capital cities in Australia.^17^ The risk factors between the populations and inadequacies in the level of medical care provided were the two main reasons that have been used to explain this excess CHD death. A recent US study showed that a mile increase in distance leads to a nearly 6.5% increase in the number of deaths from a heart attack.^18^ However in Scotland, the National Health Service is tax funded, free at the point of consumption and covers the entire population. There should thus be no socioeconomic eligibility distinctions in the level of health care given to an individual, being based on need alone. One hypothesis is that the time delay related to distance plays an important part in the prognosis of MI patients. A study from our own region demonstrated that an early thombolysis treatment strategy was achievable and improved outcome.^19^ However when we adjusted for travel time this did not explain the association between distance from acute facilities and mortality.

A US Veteran Affairs (VA) study investigated the effect of distance between home and hospital of admission on mortality in male post-MI patients.^15^ They compared the mortality outcome between patients (64%) who lived more than 20 miles from any VA health source and patients (36%) who lived within 20 miles. They found a positive relation between distance and follow-up mortality. However they studied only men and their results may not be generalisable to other healthcare systems. Our study indicates that the distance from home to hospital of admission is an important factor that predicts survival following acute MI.

### Study limitations and strengths

There are some limitations in our study. First, we did not have information on smoking, obesity, severity of MI, invasive management after discharge, the treating specialist at the admitting hospital and other unmeasured risk factors, all of which are likely to be important in patients with heart disease. However, we did use the Carstairs socioeconomic deprivation score as a surrogate measure for at least some of these factors.^20^ ^21^ Also, our previous sensitivity analysis on unmeasured risk factors showed that at least some of these unknown risk factors were unlikely to make a big impact on cardiovascular outcome.^22^ Second, the distance we used in this study was based on home and hospital addresses. We did not know the exact location of the patients when they had a heart attack. However, given the average age of 72, we would expect that the majority of patients would have had their MI at or near their home. Third, we did not have information on the time of onset of MI symptoms. Fourth, we used linear distance and estimated travel time in the study. However, distance along the road network may be different and there may have been differences in traffic conditions at different times of day.

We observed independent risk reductions in mortality during the follow-up period for lipid-lowering drugs, antiplatelet drugs and β-blockers consistent with the finding from clinical trials and other observational studies.^23^^–^28 However we may have underestimated the benefits of cardiovascular drugs because we assumed all patients complied with their drug treatment when in reality this is rarely the case.^28^ Finally, the diagnostic accuracy of MI death before hospitalisation is open to question. The data retrieved from the General Register Office revealed that in those subjects who had postmortem examinations performed, the diagnosis of MI was almost always confirmed. Presumably those subjects who underwent post mortems were those in whom the diagnosis was in question and was more likely to be incorrect. Thus these postmortem data are reassuring and suggest that prehospital MI death data were reasonably accurate.

The strength of our study is the population-based cohort design, with complete follow-up over the study period. This approach allows a “real-world” population to be studied, representing all socioeconomic groups and within a universal healthcare coverage scheme.^29^ The results inform us that there may be geographical inequality in healthcare delivery, the cause of which is currently difficult to explain. This relation may influence healthcare policy on the provision of prehospital care such as thrombolysis for MI, the enhanced provision of paramedics and the greater delivery of ambulance services to remote communities. It is in line with the recent Kerr report in Scotland calling for services to be delivered closer to patients wherever possible,^30^ and studies demonstrating that managed clinical networks can achieve this goal.^31^

In conclusion, our data suggested that distance from home to hospital may predict mortality outcome in subjects experiencing an acute MI. These data provide support for policies that locate services for acute MI closer to where patients live.
